# Herpes simplex virus 1 infection on grey matter and general intelligence in severe mental illness

**DOI:** 10.1038/s41398-022-02044-3

**Published:** 2022-07-11

**Authors:** Dimitrios Andreou, Kjetil Nordbø Jørgensen, Stener Nerland, Torill Ueland, Anja Vaskinn, Unn K. Haukvik, Robert H. Yolken, Ole A. Andreassen, Ingrid Agartz

**Affiliations:** 1 Norwegian Centre for Mental Disorders Research (NORMENT), Institute of Clinical Medicine, University of Oslo, Oslo, Norway; 2 Department of Psychiatric Research, Diakonhjemmet Hospital, Oslo, Norway; 3 Centre for Psychiatry Research, Department of Clinical Neuroscience, Karolinska Institutet & Stockholm Health Care Services, Stockholm County Council, Stockholm, Sweden; 4 Psychosis Research Section, Oslo University Hospital, Oslo, Norway; 5 Department of Psychology, University of Oslo, Oslo, Norway; 6 Norwegian Centre for Mental Disorders Research (NORMENT), Division of Mental Health and Addiction, Oslo University Hospital, Oslo, Norway; 7 Centre for Research and Education in Forensic Psychiatry, Oslo University Hospital, Oslo, Norway; 8 Stanley Division of Developmental Neurovirology, Department of Pediatrics, Johns Hopkins University School of Medicine, Baltimore, MD USA

**Keywords:** Neuroscience, Biomarkers, Schizophrenia, Bipolar disorder, Human behaviour

## Abstract

Schizophrenia and bipolar disorder are severe mental illnesses (SMI) linked to both genetic and environmental factors. Herpes simplex virus 1 (HSV1) is a common neurotropic pathogen which after the primary infection establishes latency with periodic reactivations. We hypothesized that the latent HSV1 infection is associated with brain structural abnormalities and cognitive impairment, especially in SMI. We included 420 adult patients with SMI (schizophrenia or bipolar spectrum) and 481 healthy controls. Circulating HSV1 immunoglobulin G concentrations were measured with immunoassays. We measured the total grey matter volume (TGMV), cortical, subcortical, cerebellar and regional cortical volumes based on T1-weighted MRI scans processed in FreeSurfer v6.0.0. Intelligence quotient (IQ) was assessed with the Wechsler Abbreviated Scale of Intelligence. Seropositive patients had significantly smaller TGMV than seronegative patients (642 cm^3^ and 654 cm^3^, respectively; *p* = 0.019) and lower IQ (104 and 107, respectively; *p* = 0.018). No TGMV or IQ differences were found between seropositive and seronegative healthy controls. Post-hoc analysis showed that (a) in both schizophrenia and bipolar spectrum, seropositive patients had similarly smaller TGMV than seronegative patients, whereas the HSV1-IQ association was driven by the schizophrenia spectrum group, and (b) among all patients, seropositivity was associated with smaller total cortical (*p* = 0.016), but not subcortical or cerebellar grey matter volumes, and with smaller left caudal middle frontal, precentral, lingual, middle temporal and banks of superior temporal sulcus regional cortical grey matter volumes. The results of this cross-sectional study indicate that HSV1 may be an environmental factor associated with brain structural abnormalities and cognitive impairment in SMI.

## Introduction

Schizophrenia (SZ) and bipolar disorder (BP) are severe mental illnesses (SMI) each affecting roughly 1% of the human population [1, 2]. Both genetic and environmental factors have been linked to SZ and BP [1, 2]. In the context of environmental factors, pathogens such as the neurotropic herpes simplex virus 1 (HSV1) have been implicated in both brain structural [3–6] and cognitive disturbances [7, 8] characterizing SMI.

HSV1 is a double-stranded DNA alpha herpesvirus with a high worldwide prevalence. More than half of the world’s population is HSV1-infected, with the main infection route being the salivary (oral-to-oral contact), and up to 40% of those infected develop clinical symptoms varying from frequent skin lesions to rare brain lesions [9, 10]. In particular, a high proportion of human hosts have already been HSV1-infected during childhood with the seroprevalence steadily increasing with age and reaching up to 90% in late adulthood [9]. After the primary infection the virus typically accomplishes a life-long latency in neurons which is complicated with periodic reactivations [10]. The trigeminal ganglia are thought to be the main sites of viral latency with a neuropathological study having found evidence of HSV1 DNA in the trigeminal ganglia of the majority of the HSV1 seropositive (HSV1+) individuals studied [11]. HSV1 encephalitis is an infrequent complication following mainly HSV1 reactivations but also the primary HSV1 infection [9, 12]. With the exception of encephalitis, the HSV1 exposure and the subsequent latent infection have been assumed to be benign [12]. However, magnetic resonance imaging (MRI) [3–6] and cognitive studies [7, 8] have shown aberrations in HSV1+ relative to HSV1 seronegative (HSV1−) individuals, mainly among patients with SMI, casting doubt on the supposed benign nature of the latent HSV1 infection.

Both structural and cognitive disturbances have been consistently reported in SMI. We have recently shown, studying participants drawn from the Thematically Organized Psychosis (TOP) research study cohort as in the present study, that patients with SMI display smaller total grey matter volume (TGMV) compared to healthy controls (HC) [13]. Smaller TGMV or regional grey matter volumes in SMI have also been reported by others [14–16], and include first-episode antipsychotic-naïve patients with psychosis and individuals at high genetic risk for psychosis [17]. Cognitive dysfunction is a key characteristic in SZ [18], and is frequently present before the onset of the psychotic symptoms [19], while a less severe cognitive disturbance characterizes BP [20, 21]. In a cluster analysis of the intellectual trajectories of patients with SMI, also drawn from the TOP research study cohort, Vaskinn et al. identified three cognitive subgroups: a relatively intact group (36% of the SMI sample), an intermediate group with mild cognitive impairment (44% of the SMI sample) and an impaired group with global deficits (20% of the SMI sample). All subgroups to some extent had worse cognitive performance than HC, including lower IQ for the intermediate and impaired subgroups, but for the relatively intact subgroup this was the case only for speeded neuropsychological tests [22].

The current literature on HSV1-MRI associations is inconclusive and comprises studies with small sample sizes. Studies of HSV1 and cognition have focused on specific cognitive functions, and not on general intelligence. Therefore, an examination of the association of HSV1 exposure to MRI brain measures as well as general intelligence in large well-powered studies is essential. Investigation of neuroanatomical correlates of intelligence has shown positive associations with both grey and white matter volumes with a stronger association with grey matter [23]. Further, some previous MRI studies have shown an association between HSV1 and brain structure measures among patients with SMI but not among HC [4, 6]. A susceptibility to HSV1 impact on the central nervous system may be due to immunological disturbances [24–26], an inflammatory environment or blood–brain barrier disruption in SMI [27–30]. We here hypothesized, studying a total sample of 901 participants, that HSV1+ patients with SMI will show brain structure abnormalities and cognitive impairment compared with HSV1− patients, whereas we did not expect such associations among HC. Specifically, we hypothesized that among patients with SMI, HSV1 seropositivity, reflecting previous HSV1 exposure and current viral latency, is associated with smaller TGMV volume and lower Intelligence quotient (IQ).

## Subjects and methods

### Participants

We recruited the participating patients from outpatient and inpatient psychiatric units in Oslo, Norway, as part of the TOP research study, and the HC from the same catchment area using the national population register. The TOP research study is the main study protocol at the Norwegian Centre for Mental Disorders Research (NORMENT, Oslo, Norway; www.med.uio.no/norment/english). Medical doctors and psychologists assessed the patients with the Structured Clinical Interview for DSM-IV axis I disorder (SCID-I) module A-E [31] and HC with the Primary Care Evaluation of Mental Disorders (Prime-MD) [32]. For the current study, we applied a cross-sectional approach and drew patients with SZ spectrum or BP spectrum disorders, and HC from the TOP study cohort (2005 to 2014) if HSV1, MRI and IQ data were available. We included 420 patients with SMI (239 with SZ spectrum disorders and 181 with BP spectrum disorders): 48.6% females, 46.9% HSV1 seropositivity, mean age (SD) = 32.5 (10.5), and 481 HC: 45.3% females, 45.1% HSV1 seropositivity, mean age (SD) = 34 (9.1). Specifically, we included patients with SZ (*n* = 125), schizophreniform disorder (*n* = 21), schizoaffective disorder (*n* = 29), delusional disorder (*n* = 10), brief psychotic disorder (*n* = 8), psychotic disorder not otherwise specified (NOS) (*n* = 46), BP I (*n* = 103), BP II (*n* = 70) and BP NOS (*n* = 8). We applied the following exclusion criteria for patients and HC: previous moderate or severe head injury, a neurological disorder or medical conditions that could affect brain function. None of the participating patients or HC had a history of encephalitis, an infrequent complication of HSV1 infection [12]. We further excluded HC with previous or current psychiatric disorders including substance use disorders (including alcohol use disorder) or with close relatives with SMI. The criteria for inclusion and exclusion were pre-established.

The study was approved by the Regional Committee for Medical Research Ethics South East Norway (REC South East) and the Norwegian Data Inspectorate, and was conducted in accordance with the Declaration of Helsinki as revised in 2008. We obtained written informed consent from all participants.

Data supporting the findings of the present study have repository at NORMENT/Oslo University Hospital. Restrictions apply to the availability of data and are thereby not publicly available. Data can be made available under reasonable request and with permission of NORMENT/Oslo University Hospital, in accordance with the ethics agreements/research participants consent.

### Measures and medication variables

Education level (or education years) has been largely used as a socioeconomic status indicator capturing the shift from parental to individual socioeconomic status [33]. We used years of education as proxy indicator for socioeconomic status. Further, we assessed current IQ with a licensed translated version of the Wechsler Abbreviated Scale of Intelligence (WASI) [34]. We assessed alcohol use with the alcohol use disorder identification test (AUDIT) [35] and drug use with the drug use disorder identification test (DUDIT) [36]. We evaluated patients’ severity of illness with the Global Assessment and Functioning (GAF) scale [37] and the Positive and Negative Syndrome Scale (PANSS) [38] and defined the duration of illness (DOI) as time passed from the first psychotic episode for patients with SZ spectrum disorders and from the first affective episode for patients with BP spectrum disorders. We finally obtained information of current use of antipsychotics, antidepressants, antiepileptics and lithium (binary variables, yes/no).

### Brain MRI acquisition and analysis

901 T1-weighted MRI scans were obtained: 554 on a 1.5 T Siemens MAGNETOM Sonata scanner with a standard head coil, and 347 scans on a 3 T General Electric Signa HDxt scanner with an 8-channel head coil. T1-weighted sequences, Siemens 1.5 T Magnetom Sonata scanner**:** A sagittal magnetization prepared rapid gradient echo (MPRAGE) sequence was used to acquire two T1-weighted volumes during the same scan session with the following parameters: Echo time (TE) = 3.93 ms, repetition time (TR) = 2730 ms, inversion time (TI) = 1000 ms, flip angle = 7°; Field of view (FOV) = 24 cm, voxel size = 1.33 × 0.94 × 1 mm, number of partitions = 160. The two volumes obtained were averaged during post-processing to increase signal-to-noise ratio (SNR). T1-weighted sequences, General Electric 3 T Signa HDxt scanner**:** A 3D fast spoiled gradient echo (FSPGR) sequence was used to acquire T1-weighted volumes using the following parameters: Echo time (TE) = MinFull, repetition time (RT) = 7.8 ms, inversion time (TI) = 450 ms, Field of view = 256 × 256 mm, voxel size = 1x1x1.2 mm, flip angle = 12°, 170 sagittal slices.

MRI scans were processed using the FreeSurfer v6.0.0 [39]. TGMV was calculated as the sum of the cortical, subcortical and cerebellar grey matter volumes (https://surfer.nmr.mgh.harvard.edu/fswiki/MorphometryStats). We obtained regional cortical volumes based on the Desikan-Killiany FreeSurfer Atlas [40]. Quality inspection and editing was performed by trained research assistants following standard FreeSurfer procedures [41].

### Serology assessment

Blood samples were drawn from all participants. Serology assessment was performed at the Stanley Neurovirology Laboratory (Johns Hopkins University School of Medicine, Baltimore, MD, USA). HSV1 immunoglobulin G (IgG) concentrations were measured by solid-phase immunoassay techniques directed at the HSV1 specific gG1 glycoprotein, and expressed as dichotomous measures (seropositivity/seronegativity), derived via comparisons of the reactivity generated by the samples in the immunoassay with the optical density generated by standard samples as previously described; for the post-hoc analyses the continuous HSV1 IgG concentration variable was also used [42].

### Statistics

#### Main analysis

In the bivariate analysis among HC (*n* = 481), applying chi-square tests for categorical variables and *t*-tests for quantitative variables, we assessed group differences between HSV1+ and HSV1− HC in sex, age, education years, handedness, AUDIT score, DUDIT score and the estimated total intracranial volume (ICV). Similarly, in the bivariate analysis among patients with SMI (*n* = 420), we assessed group differences between HSV1+ and HSV1− participants in the same variables as in HC as well as the following patient-related variables: DOI, PANSS total score, GAF-symptoms and GAF-functioning scores, and the current use of antipsychotics, antidepressants, antiepileptics and lithium.

In the main multivariate models (analyses of covariance; ANCOVAs), among patients and HC separately, we investigated the main effects of HSV1 status (HSV1+/HSV1−) on TGMV controlling for sex, age and scanner, as well as on IQ controlling for sex and age. In the case of significant group differences in the bivariate analysis (Table 1), we aimed to run additional ANCOVAs also controlling for variables that significantly differed between HSV1+ and HSV1− participants.

**Table 1:** Group differences between herpes simplex virus 1 (HSV1) immunoglobulin G (IgG) seropositive (HSV1+) and seronegative (HSV1−) patients with severe mental illness (SMI) in sex, age, education years, handedness (right-handedness vs. left-handedness/ambidexterity), duration of illness (DOI), Positive and Negative Syndrome Scale (PANSS) total score, the Global Assessment and Functioning-symptoms (GAF-S) and GAF-functioning (GAF-F) scores, the percentage of patients on antipsychotics, antidepressants, antiepileptics and lithium, alcohol use disorder identification test (AUDIT) score, drug use disorder identification test (DUDIT) score and estimated total intracranial volume (ICV). Group differences between HSV1+ and HSV1− healthy controls (HC) in sex, age, education years, handedness, AUDIT score, DUDIT score and ICV.

	HSV1+		HSV1−		
	N^a^	Mean (SD) or %	N^a^	Mean (SD) or %	*P* value^b^
Patients with SMI					
Sex (% women)	197	46.7	223	50.2	0.471
Age (years)	197	33.4 (10.5)	223	31.6 (10.4)	0.068
Education years	196	13 (2.5)	223	13.7 (2.5)	0.766
Handedness (% right-handedness)	196	87.3	223	89.2	0.527
DOI (years)	195	10.8 (9.2)	218	9.3 (8.8)	0.093
PANSS total score^c^	194	53.2 (15.3)	222	53.3 (16.9)	0.963
GAF-S	197	50 (13.2)	223	50.3 (13.2)	0.781
GAF-F	197	49.2 (12.8)	223	50 (13)	0.557
On antipsychotics (%)	197	74.1	223	70.4	0.398
On antidepressants (%)	197	33	223	35.4	0.600
On antiepileptics (%)	197	29.4	223	21.5	0.062
On lithium (%)	197	9.1	223	9.9	0.800
AUDIT score	139	8.1 (7.3)	149	7.8 (6.2)	0.721
DUDIT score	144	4.3 (8)	155	3.9 (7.7)	0.691
ICV (cm^3^)	197	1568 (182)	223	1590 (168)	0.192
HC					
Sex (% women)	217	44.2	264	46.2	0.665
Age (years)	217	34.9 (8.3)	264	33.3 (9.7)	**0.012**^d^
Education years	217	14.4 (2.3)	264	14.4 (2.2)	0.808
Handedness (% right-handedness)	217	88.9	264	89.4	0.873
AUDIT score	122	5.7 (3.6)	155	5.6 (3)	0.671
DUDIT score	128	0.4 (1.8)	154	0.2 (0.9)	0.241^d^
ICV (cm^3^)	217	1558 (168)	264	1567 (161)	0.575

*P* values < 0.05 shown in bold.

^a^Number of participants with data in each variable.

^b^Chi-square test or *t*-test.

^c^Separate analysis for SZ and BP spectrum disorders showed that HSV1+ and HSV1− patients did not differ in PANSS total score in either diagnostic group (*p* = 0.949 and 0.537 for SZ and BP spectrum disorders, respectively).

^d^Mann–Whitney *U* test.

#### Post-hoc analysis among patients with severe mental illness

In order to follow up the significant HSV1-TGMV association among patients with SMI (described in the results section) we divided the TGMV into its three components (cortical, subcortical and cerebellar grey matter volumes), ran sex-, age- and scanner-adjusted ANCOVAs and accepted a Bonferroni-corrected alpha level of 0.05/3 = 0.017. In order to follow up the significant HSV1-cortical grey matter volume association (described in the results section), we divided the cortical grey matter volume into 34 left and 34 right regional cortical volumes based on the Desikan-Killiany FreeSurfer Atlas [40], ran 34 sex-, age- and scanner-adjusted ANCOVAs by hemisphere and applied a false discovery rate (FDR) of 5% by hemisphere to correct for multiple testing [43]. For significant HSV1-regional cortical volumes associations (described in the results section): (1) We ran ANCOVAs correcting for ICV. (2) We ran sex-, age-, and scanner-adjusted multiple regressions investigating the associations between HSV1 concentrations and the regional cortical volumes. (3) To determine SMI specificity, we included all patients and HC (*n* = 901) and ran sex-, age- and scanner-adjusted ANCOVAs including the HSV1 status-by- patient/control interaction term.

All tests were two-sided. We conducted all the analyses with IBM SPSS Statistics 28.

## Results

### Total grey matter volume analysis

#### Patients with severe mental illness

**Fig. 1:**
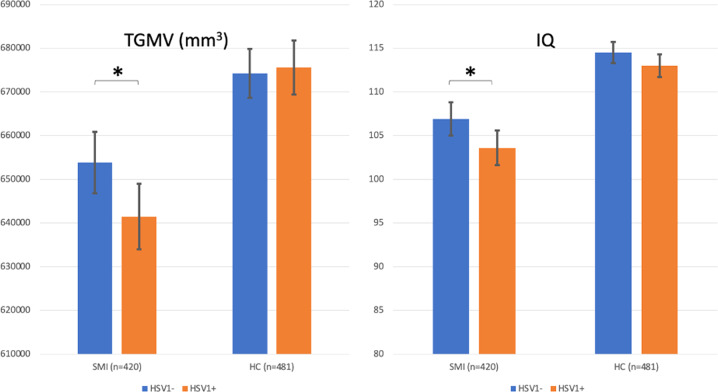
TGMV and IQ in HSV1+ and HSV1− patients with SMI and HC. Left: Total grey matter volume (TGMV) in mm^3^ in herpes simplex virus 1 (HSV1) immunoglobulin G (IgG) seropositive (HSV1+) and seronegative (HSV1−) patients with severe mental illness (SMI) and healthy controls (HC). Right: Intelligent quotient (IQ) in HSV1 + and HSV1− patients with SMI and HC. HSV1+ patients had significantly smaller TGMV and lower IQ compared with HSV1− patients, whereas no such differences were found between HSV1+ and HSV1− HC. 420 patients (223 HSV1−/197 HSV1+) and 481 HC (264 HSV1− and 217 HSV1+) were included in both analyses. Adjusted means with 95% confidence intervals are shown *p < 0.05.

The bivariate analysis among patients with SMI showed that HSV1+ patients did not significantly differ from HSV1− patients in any of the analyzed variables (Table 1). In the sex-, age- and scanner-adjusted multivariate model (ANCOVA) (*n* = 420), there was a significant main effect of HSV1 status on TGMV, *F*(1,415) = 5.557, *p* = 0.019, partial eta-squared = 0.013, with HSV1+ patients having smaller TGMV than HSV1− patients, a significant main effect of sex (*p* < 0.001), with women having smaller TGMV than men, a significant main effect of age (*p* < 0.001), with age being inversely associated with TGMV as well as a significant main effect of the scanner variable (*p* < 0.001). The estimated TGMV marginal means were 642 cm^3^ and 654 cm^3^ for HSV1+ and HSV1− patients, respectively (Fig. 1).

#### Healthy controls

The bivariate analysis among the HC showed that HSV1+ HC were 1.6 years older than HSV1− HC assessed with *t*-test (*p* = 0.049) but did not significantly differ in any other of the analyzed variables (Table 1). In the multivariate sex-, age- and scanner-adjusted model (ANCOVA) (*n* = 481), we did not find any main effect of HSV1 status on TGMV, *F*(1,476) = 0.100, *p* = 0.752, partial eta-squared <0.001, while sex, age and scanner were all significantly associated with TGMV as in the patient group (*p* < 0.001 for all three). The estimated TGMV marginal means were 676 cm^3^ and 674 cm^3^ for HSV1+ and HSV1− HC, respectively (Fig. 1).

### IQ analysis

#### Patients with severe mental illness

In the sex- and age-adjusted model (ANCOVA) (*n* = 420), there was a significant main effect of HSV1 status on IQ, *F*(1,416) = 5.689, *p* = 0.018, partial eta-squared = 0.013, with HSV1+ patients having lower IQ than HSV1− patients, and no main effect of sex (*p* = 0.637) or age (*p* = 0.150) on IQ. The estimated IQ marginal means were 103.6 and 106.9 and for HSV1+ and HSV1− patients, respectively (Fig. 1).

#### Healthy controls

In the sex- and age-adjusted model (ANCOVA) (*n* = 481), we did not find any main effect of HSV1 status on IQ, *F*(1,477) = 2.568, *p* = 0.104, partial eta-squared = 0.006, sex was associated with IQ (*p* = 0.015), with women having lower IQ than men, while age was not associated with IQ (*p* = 0.167). The estimated IQ marginal means were 113 and 114.5 for HSV1+ and HSV1− HC, respectively (Fig. 1).

### Post-hoc analyses

#### Analysis of cortical, subcortical and cerebellar grey matter volumes

**Fig. 2:**
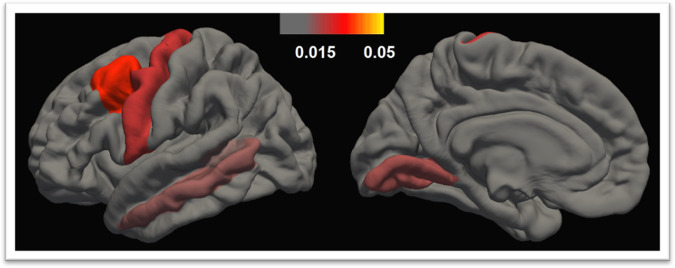
Cortical regional volumes associated with HSV1 seropositivity in SMI. Herpes simplex virus 1 (HSV1) immunoglobulin G (IgG) seropositive (HSV1+) patients with severe mental illness compared to seronegative (HSV1−) patients displayed significantly smaller (after false discovery rate correction of 5% by hemisphere) left caudal middle frontal, left precentral, left lingual, left middle temporal and left banks of superior temporal sulcus volumes. Color bar represents effects sizes: the variation in regional volumes explained by HSV1 status (partial eta^2^ derived from the sex- age- and scanner-adjusted analyses of covariance).

To follow up the significant HSV1-TGMV association among patients with SMI, we investigated the putative associations between HSV1 status and the cortical, the subcortical and the cerebellar grey matter volumes (*n* = 420 for all the analyses). We ran three sex-, age- and scanner-adjusted ANCOVAs and found a significant (corrected alpha level 0.05/3 = 0.017) inverse association between HSV1 status and the cortical grey matter volume, *F*(1,415) = 5.849, *p* = 0.016, partial eta-squared=0.014, but not the subcortical, *F*(1,415) = 1.367, *p* = 0.243, partial eta-squared = 0.003, or the cerebellar grey matter volume, *F*(1,415) = 1.898, *p* = 0.169, partial eta-squared = 0.005. To follow up the HSV1-cortical grey matter volume association, we investigated the putative associations between HSV1 status and all 34 left and 34 right regional cortical volumes [40]. After FDR correction, HSV1+ patients compared with HSV1− patients demonstrated significantly smaller left caudal middle frontal, left precentral, left lingual, left middle temporal and left banks of superior temporal sulcus volumes (Table 2 and Fig. 2). The associations survived ICV correction (Table 2). Further, as shown in Table 2, HSV1 antibody levels were inversely associated with these five regional cortical volumes.

**Table 2:** In the analysis of herpes simplex virus 1 (HSV1) immunoglobulin G (IgG) status (HSV1 antibody positivity/negativity; HSV1+/HSV1−) on regional cortical volumes among patients with severe mental illness (*n* = 420), we ran 34 sex-, age- and scanner-adjusted analyses of covariance (ANCOVAs) by hemisphere.

	ANCOVAs (HSV1+/HSV1−)				ICV-corrected ANCOVAs	Multiple regressions (HSV1 levels)
	Direction	*p* values	*q* values	Partial eta^2^	*p* values	*p* values
Left regional cortical volumes						
Caudal middle frontal	–^a^	0.0001^b^	0.005	0.034	0.001	0.003
Precentral	–	0.001^b^	0.017	0.025	0.007	0.003
Lingual	–	0.003^b^	0.034	0.021	0.014	0.002
Middle temporal	–	0.005^b^	0.043	0.019	0.033	0.027
Banks of superior temporal sulcus	–	0.007^b^	0.048	0.017	0.037	0.002
Supramarginal	–	0.015	ns^c^			
Postcentral	–	0.019	ns			
Rostral anterior cingulate	–	0.026	ns			
Frontal pole	–	0.044	ns			
Right regional cortical volumes						
Lingual	–	0.007	ns			
Pars opercularis	–	0.008	ns			
Precentral	–	0.015	ns			
Paracentral	–	0.023	ns			
Caudal middle frontal	–	0.036	ns			
Superior frontal	–	0.036	ns			
Frontal pole	–	0.038	ns			
Fusiform	–	0.044	ns			
Postcentral	–	0.044	ns			

The *p* values of all nominally significant associations are presented. Applying a false discovery rate (FDR) of 5% by hemisphere to correct for multiple testing, HSV1+ patients had significantly smaller left caudal middle frontal, left precentral, left lingual, left middle temporal and left banks of superior temporal sulcus volumes compared with HSV1− patients. For the significant associations *q* values, effect sizes (partial eta^2^), *p* values from sex-, age-, scanner- and estimated intracranial volumes (ICV)- corrected ANCOVAs as well as *p* values from sex-, age- and scanner-adjusted multiple regressions of the HSV1 levels on regional volumes are presented.

^a^Smaller regional volumes in HSV1+ patients compared with HSV1− patients.

^b^Survives FDR correction.

^c^Non-significant.

Finally, to determine SMI specificity, we included all patients and HC (*n* = 901) and ran sex-, age-, and scanner-adjusted ANCOVAs investigating HSV1-by-patient/control status interactions. Significant interactions were found in the models on the cortical grey matter volume, left caudal middle frontal, left precentral, left lingual and left banks of superior temporal sulcus volumes (*p* values for the interaction terms were 0.036, <0.001, 0.024, 0.024 and <0.001, respectively). In the model on the left middle temporal volume, the interaction was not statistically significant (*p* = 0.088).

#### Total grey matter volume and IQ analysis in diagnostic subgroups

HSV1+ and HSV1− patients in the SZ spectrum or the BP spectrum did not significant differ in any of the analyzed variables (Suppl. Table 4). We investigated the main effect of HSV1 IgG status on TGMV, whilst controlling for age, sex and scanner, in patients with SZ spectrum (*n* = 239) and BP spectrum disorders (*n* = 181) separately. In both patient groups, HSV1 + patients had similarly non-significantly smaller TGMV volumes compared to HSV1− patients, *F*(1,234) = 2.861, *p* = 0.092, partial eta-squared = 0.012, and *F*(1,176) = 2.077, *p* = 0.151, partial eta-squared = 0.012, in SZ and BP spectrum, respectively. In SZ spectrum, the estimated TGMV marginal means were 641 cm^3^ and 654 cm^3^ for HSV1+ and HSV1− patients, respectively, while in BP spectrum, the estimated TGMV marginal means were 643 cm^3^ and 653 cm^3^ for HSV1+ and HSV1− patients, respectively. We investigated the main effect of HSV1 IgG status on IQ, whilst controlling for age and sex, in patients with SZ spectrum (*n* = 239) and BP spectrum disorders (*n* = 181) separately. In SZ spectrum, HSV1+ patients had significantly lower IQ compared to HSV1− patients, *F*(1,235) = 5.820, *p* = 0.017, partial eta-squared = 0.024, whereas in BP spectrum, HSV1+ patients did not differ from HSV1− patients, *F*(1,177) = 0.129, *p* = 0.720, partial eta-squared = 0.001. In SZ spectrum, the estimated IQ marginal means were 100.1 and 104.8 in HSV1+ and HSV1− patients, respectively. In BP spectrum, the estimated IQ marginal means were 108.7 and 109.3 in HSV1+ and HSV1− patients, respectively.

## Discussion

In the present study we showed that HSV1+ patients with SMI had significantly smaller TGMV as well as lower IQ than HSV1− patients (Fig. 1). There were no TGMV or IQ differences between HSV1+ and HSV1− HC (Fig. 1). Further, among both patients with SMI and HC, women had smaller TGMV than men while age was inversely associated with TGMV (Suppl. Table 2) in line with previous reports [13, 44, 45]. Our post-hoc analysis among patients with SMI showed that HSV1 seropositivity was associated with smaller cortical grey matter volume (but not subcortical or cerebellar grey matter volumes), mainly with smaller caudal middle frontal, precentral, lingual, middle temporal and banks of superior temporal sulcus volumes, all in the left hemisphere (Table 2 and Fig. 2). Of note, all five regional volumes survived ICV correction (Table 2) while the cortical grey matter volume and four out of the five regional volumes (all but the left middle temporal volume) showed SMI specificity determined by significant HSV1 status-by-patient/control status interactions. Finally, HSV1 antibody levels were inversely associated with all five left regional cortical volumes (Table 2). The interpretation of the continuous IgG antibody levels against Herpesviridae is not established; higher concentrations might indicate more frequent reactivations [46, 47].

The putative associations between HSV1 seropositivity and brain structure measures in SZ have been investigated in a few previous studies with small sample sizes (26–40 patients with SZ) whereas, to our knowledge, such associations have not been investigated in BP. Pandurangi et al. included medicated adult patients with SZ and reported diffuse structural disturbances including cortical atrophy, smaller left frontal area and callosal aberrations [3]. Schretlen et al. studied adult patients with SZ and found (non-significantly, *p* = 0.058) smaller TGMV in HSV1+ patients than in HSV1− patients [5]. Our results showing smaller total and cortical grey matter volumes in HSV1+ patients are in line with those previous studies showing cortical atrophy and smaller TGMV. Of note, in our study the effect sizes were rather small, and the significant inverse HSV1 seropositivity-TGMV association was found only in the larger sample of all patients (*n* = 420), and not in the smaller samples of patients with SZ (*n* = 239) or BP (*n* = 181) suggesting that larger samples of SZ and BP patients need to be studied.

Regional grey matter volumes have also been investigated in SZ. In particular, Schretlen et al. reported smaller grey matter volumes in the anterior cingulate cortex and the cerebellum in HSV1+ adult patients with SZ compared with HSV1− patients [5]. Prasad et al. studied first-episode antipsychotic-naïve patients with SZ spectrum disorders and HC, and reported smaller grey matter volumes in the dorsolateral prefrontal cortex and the anterior cingulate cortex in HSV1+ patients relative to HSV1− patients, while there was no such difference in HC [4]. We found a nominally significant association between HSV1 seropositivity and the left rostral anterior cingulate volume (Table 2) which is in line with those previous reports. As shown in Table 2 and Fig. 2, our results are also indicative, partially in line with the report by Prasad et al., of a frontal lobe susceptibility (two out of the five significant associations and eight out of the 18 nominally significant associations concerned frontal cortical regions). In addition, a longitudinal study of patients with SZ and HC showed a significant grey matter loss in the posterior cingulate cortex in HSV1+ patients with SZ over one year but not in HSV1− patients with SZ, HSV1 + HC or HSV− HC [6]. Finally, studying HSV1+ and HSV1− individuals with a high risk of developing psychosis and HC, Whitford et al. reported smaller cuneus in the HSV1+ group compared with both the HSV1− group and the HC [48].

Stratifying by diagnostic group, HSV1 seropositivity was similarly (non-significantly) associated with smaller TGMV in both patients with SZ spectrum and BP spectrum disorders, whereas HSV1 seropositivity was associated with lower IQ in SZ spectrum, but not in BP spectrum. These results suggest that HSV1 infection is linked to smaller TGMV in SMI irrespective of specific diagnosis, and to lower general intelligence in SZ spectrum disorders. The interpretation of the HSV1-IQ association in SZ but not in BP spectrum disorders despite a similar impact on TGMV is necessarily speculative. Deleterious genetic or environmental influence in SZ or the lack of such influence in BP might explain the discrepancy. The two disorders have both shared and independent genetic influences [49]. Further, most SZ risk alleles are linked to lower intelligence, whereas most BP risk alleles are linked to higher intelligence [50]. The two disorders have also different cognitive profiles with patients with BP showing a less severe cognitive dysfunction [20, 21]. Compared with SZ where cognitive abnormalities are often already present before the emergence of psychotic symptoms [19], in BP, higher and lower premorbid cognitive performances have both been linked to higher risk of developing BP [51–53].

The association between HSV1 seropositivity and cognitive function has been previously explored in SZ and BP as well as in HC. In a recent review and meta-analysis, HSV1+patients with SMI (SZ or BP) had worse cognitive performance compared with HSV1− patients, with both patient groups (HSV1+ and HSV1−) performing worse than HC [8]. Six out of the included nine studies and three out of the included four studies showed significant associations between HSV1 seropositivity and impaired cognition among patients with SZ and BP, respectively [8]. Interestingly, in line with our results in SZ, the results of two studies combined showed that HSV1+ patients had worse cognitive functioning than HSV1− patients measured with the Repeatable Battery for the Assessment of Neuropsychological Status (RBANS) score [8], an index highly correlated with IQ in this patient group [54]. Further, in another recent meta-analysis of more than 3500 patients with SZ from nine studies, HSV1 seropositivity was also significantly associated with cognitive impairment [7].

The putative association between HSV1 seropositivity and cognitive functioning in the general population (not selecting on mental health status) or among HC has been investigated in previous studies with conflicting results [55–58]. In line with the present results showing lack of association in HC, a longitudinal analysis of a large sample of elderly adults (*n* = 1204) failed to find any association between HSV1 antibody levels and cognitive functioning at baseline or cognitive decline at 4-years follow-up [57]. However, in another large population-based study, HSV1 seropositivity was associated with worse cognitive functioning among children (*n* = 1419), non-elderly adults (*n* = 3816) and elderly adults (*n* = 2394) [58]. In a mixed sample of adult patients with SZ, their relatives and HC (*n* = 1852), HSV1 seropositivity was inversely associated with cognitive functioning [56]. These findings among adults may be explained by the inclusion of patients with history of psychiatric disorder, but this is a less plausible explanation among children where a history of psychiatric disorder, especially SMI, is far less prevalent. In addition, even when adults without a history of psychiatric disorders (*n* = 240) were studied, HSV1 seropositivity was associated with worse cognitive performance [55]. The discrepancy may be due to different cognitive measures or populations studied. We have studied a large sample of non-elderly adult HC (*n* = 480), and did not find any association between HSV1 seropositivity and IQ (or MRI brain measures) diminishing the possibility of a harmful impact of HSV1 on general intelligence in adults without history of psychiatric disorders.

We have shown that HSV1 seropositivity reflecting previous infection and current latency is associated with cognitive as well as brain structure abnormalities. The HSV1 latent infection is more active than previously thought, and is characterized by a high expression of latency-associated transcripts [59]. The observed association between HSV1 seropositivity and TGMV loss may be a result of the primary infection, the viral reactivations or the chronic latent infection. The neuropathological aberrations underlying the HSV1-related grey matter volume loss might be neuronal loss or neuronal size reduction [12]. Further, our results are indicative of a susceptibility to HSV1 effects on brain structure and cognition in patients with SMI, which was not found in HC. SMI-related immune response or blood–brain barrier abnormalities may render the brain of patients particularly vulnerable to HSV1. By contrast, HC appear resilient which may be due to a sufficient immune response against HSV1 and a blood–brain barrier integrity.

Compared with HSV1− patients, HSV1+ patients had significantly smaller regional cortical volumes in the left frontal (caudal middle frontal and precentral), temporal (middle temporal and banks of superior temporal sulcus) and occipital (lingual) lobes (*p*_FDR_ < 0.05) (Table 2 and Fig. 2). Further, HSV1+ patients had nominally significantly (*p*_uncorrected_ < 0.05) smaller cortical volumes in totally nine left and nine right regions, predominately in the frontal lobes (Table 2). In a recent meta-analysis of almost 10000 individuals, patients with SZ compared to HC demonstrated smaller regional cortical thickness and surface areas, which are the two components of the cortical volumes, with the largest effect sizes for frontal and temporal regions [60]. Taken together, HSV1 infection appears linked to brain regions that are predominately affected in psychosis, and although our results cannot determine causality, we are tempted to speculate that HSV1 may be an environmental factor playing a role in brain abnormalities found in SMI. Intriguingly, the temporal and frontal brain regions are also those predominately affected in HSV1 encephalitis, an infrequent HSV1 complication with a high mortality rate and dramatic symptomatology including neurological sequalae, cognitive impairment and psychotic symptoms [12]. The frontal cortex has also been repeatedly implicated in general intelligence followed by temporal, parietal and occipital regions [23, 61].

Even though the effect sizes were small for the TGMV and the total cortical volume analyses, they were larger for the regional volume analyses (left caudal middle frontal, precentral, lingual, middle temporal and banks of superior temporal sulcus cortical volumes) with HSV1 status explaining up to 3.4% of the variation in the regional volumes (Table 2). Of note, in our whole sample analysis (Suppl. Table 3), patients with SMI had smaller TGMV, total cortical volume and 4/5 regional volumes (left caudal middle frontal, precentral, lingual and middle temporal) with the SMI/HC status explaining up to 1.3% of the variation in these brain volumes. This may indicate the clinical importance of the HSV1 effect, being at least similar to the SMI effects on the brain volumes analyzed.

Taken together, HSV1-exposed patients with SMI demonstrate smaller TGMV relative to non-exposed patients, and for patients with SZ a HSV1-related cognitive disturbance is also evident. A plausible interpretation of the current results could be that patients with SMI who contract HSV1, before or after the disorder onset, develop a more severe form of the disorder with smaller TGMV and for SZ even worse cognitive performance. An alternative interpretation is that HSV1 infection leads to TGMV loss (and lower IQ in SZ) which then predisposes individuals to SMI and thus the association between HSV1 and these mediators is most predominant in those with SMI. This is supported by the fact that regional grey matter volumes that are smaller in patients than in HC and in HSV1+ patients than in HSV1- patients partially coincide. However, such an etiological HSV1 role is not supported by the absence of a higher frequency of HSV1 seropositivity in patients than in HC (suppl. material).

The study has certain limitations. First, it has a cross-sectional design and causality cannot be determined. Further, despite the fact that we have accounted for putative confounders, including age, years of education as a proxy for socioeconomic status, handedness, DOI, substance use including alcohol as well as medication use, we cannot exclude that other unknown factors may influence the associations between CMV and brain structural measures or IQ. A related limitation is that we had data on current but not lifetime medication use. Further, we cannot determine when the primary HSV1 infection and the subsequent reactivations took place. Even though seropositivity typically reflects current latency, we cannot know whether it is the primary infection, the viral reactivations or the non-silent ongoing latent infection that are accountable for the observed smaller brain structures and lower IQ. Another limitation is that we have analyzed all patients with SZ spectrum disorders as one group, and similarly, all patients with BP spectrum disorders as one group. Future studies with larger samples could study individual diagnoses within the SZ and BP spectrum. Finally, all the diagnoses were lifetime diagnoses based on the SCID-I interviews and may not reflect the current state of the participating patients.

To conclude, HSV1 seropositivity in SMI, but not among HC, was linked to both smaller TGMV and lower IQ. HSV1 seropositivity in SMI was also inversely associated with the total and regional cortical grey matter volumes mainly in frontal and temporal lobes of the left hemisphere. The results suggest that patients with SMI who have contracted HSV1 infection develop a disorder with augmented structural and cognitive aberrations compared to patients that have not been exposed to HSV1.

## Supplementary information

Suppl. Material
