# Supplementary material for: Herpes simplex virus 1 infection on grey matter and general intelligence in severe mental illness
Source: Transl Psychiatry. 2022 Jul 11;12:276. doi: 10.1038/s41398-022-02044-3 (PMC9276804; doi:10.1038/s41398-022-02044-3)
Supplement: Supplementary file 1 — Suppl. Material [file 41398_2022_2044_MOESM1_ESM.docx]

**Supplementary material**

***Power analysis***

In our previous studies on human herpes virus 5 (HHV5/cytomegalovirus) on brain measures and general intelligence, the effect sizes were small to medium (partial eta^2^ 0.03-0.06) ^1,2^. In the present study, the sample size to ensure adequate power to detect such an effect size (partial eta^2^ 0.03) is 423 (partial eta^2^=0.03, effect size f=0.1758631, a err prob = 0.05, power = 0.95) ^3^. We have included 420 patients with SMI and 481 HC ensuring adequate power in both groups.

***Included vs not included participants***

In the Thematically Organized Psychosis (TOP) research study cohort, 1010 patients with SMI had HSV1 data. 420/1010 patients had both MRI and IQ data and were included in the present study. Similarly, 702 HC had HSV1 data. 481/701 had both MRI and IQ data and were included in the present study. Included and non-included patients did not differ in the HSV1 seropositivity, sex or age. Included and non-included HC did not differ in HSV1 seropositivity or sex, while included HC were 2 years younger than non-included HC (p=0.010).

|  | **Patients with SMI** | | | | | **Healthy controls** | | | | |
| --- | --- | --- | --- | --- | --- | --- | --- | --- | --- | --- |
|  | **Included** | | **Not included** | |  | **Included** | | **Not included** | |  |
|  | **N^1^** | **Mean (SD) or %** | **N^1^** | **Mean (SD) or %** | **P value^2^** | **N^1^** | **Mean (SD) or %** | **N^1^** | **Mean (SD) or %** | **P value^2^** |
| HSV1 seropositivity | 420 | 46.9 | 590 | 47.8 | 0.780 | 481 | 45.1 | 221 | 51.1 | 0.138 |
| Sex (% women) | 420 | 48.6 | 590 | 47 | 0.611 | 481 | 45.3 | 221 | 51.6 | 0.123 |
| Age  (years) | 420 | 31.4 (10.4) | 590 | 31.9 (10.8) | 0.465 | 481 | 32.9  (8.8) | 213 | 35  (10.8) | **0.010** |

**Suppl. Table 1.** Group differences between included and not included patients with severe mental illness (SMI), and included and not included healthy controls in herpes simplex virus 1 (HSV1) immunoglobulin G (IgG) seropositivity, sex and age. P values <0.05 shown in bold

^1^Number of participants with data in each variable

^2^Chi-square test or t-test

***Sex, age and HSV1 status in patients with SMI and HC***

HC and patients with SMI did not significantly differ in sex (45.3% and 48.6% females among HC and patients, respectively; p=0.330) or HSV1 seropositivity (45.1% and 46.9% in HC and patients, respectively; p=0.591), assessed with chi-square tests. HC were 1.5 years older than patients with SMI (34 and 32.5 years for HC and patients, respectively; p=0.019), assessed with t-test.

***MRI analysis***

*Patients with severe mental illness*

There was homogeneity of variances evaluated with Levene’s test, p=0.959. The residuals of the overall model were approximately normally distributed determined by visual inspection. There were two outliers with studentized residuals greater than three standard deviations. These cases did not exhibit high leverage (0.07 and 0.01 for case one and two, respectively) or high Cook's distance value (0.02 for both cases) and were included in the final model. In a model where these cases were excluded, there was still as a significant main effect of HSV1 status on TGMV (p=0.040).

*Healthy controls*

There was homogeneity of variances evaluated with Levene’s test, p=0.328. The residuals of the overall model were approximately normally distributed determined by visual inspection. There were two outlier with studentized residuals greater than three standard deviations. These cases did not exhibit high leverage (0.01 for both cases) or high Cook's distance value (0.03 and 0.02 for case one and two, respectively) and were included in the final model. In a model where these cases were excluded, there was still no main effect of HSV1 status on TGMV (p=0.735).

*Selection of post-hoc analyses*

The selection of the post-hoc analyses was based on the significant associations found. We conducted post-hoc analyses in patients due to the significant association found in the main analysis (HSV1-TGMV), whereas we did not conduct any post-hoc analyses for HC as there was no association in the main analysis (HSV1-TGMV). Due to the significant HSV1-TGMV association among patients we then analyzed the cortical, subcortical and cerebellar grey matter volumes, and due to the significant HSV1-cortical grey matter volume association, we analyzed all the cortical regional volumes. Due to the lack of a HSV1-subcortical grey mater association we did not analyze the subcortical structures.

*Sex and age on TGMV*

|  | P-value | Partial eta^2^ |
| --- | --- | --- |
| **Whole sample (N=901)** |  |  |
| Sex | <0.001 | 0.339 |
| Age | <0.001 | 0.148 |
| Scanner | <0.001 | 0.159 |
| **Scanner 1 (N=554)** |  |  |
| Sex | <0.001 | 0.339 |
| Age | <0.001 | 0.155 |
| **Scanner 2 (N=347)** |  |  |
| Sex | <0.001 | 0.340 |
| Age | <0.001 | 0.137 |
| **Scanner 1, SMI (N=334)** |  |  |
| Sex | <0.001 | 0.291 |
| Age | <0.001 | 0.132 |
| **Scanner 1, HC (N=220)** |  |  |
| Sex | <0.001 | 0.445 |
| Age | <0.001 | 0.245 |
| **Scanner 2, SMI (N=86)** |  |  |
| Sex | <0.001 | 0.218 |
| Age | <0.001 | 0.164 |
| **Scanner 2, HC (N=261)** |  |  |
| Sex | <0.001 | 0.386 |
| Age | <0.001 | 0.131 |

**Suppl. Table 2.** The p-values and the corresponding partial eta-squared of the analyses of covariance (ANCOVAs) exploring sex and age effects on total grey matter volume (TGMV) in the whole sample as well as in subgroups after stratification by scanner and patient-control status

*Patient-control analysis on TGMV and cortical volumes*

In the whole sample (n=901), we ran additional analyses to explore the putative patent/control differences in TGMV and the cortical volumes that significantly differed between HSV1+ and HSV1- patients (Table 2) (the total cortical volume and five left regional cortical volumes). In sex-, age- and scanner-adjusted multivariate models (ANCOVAs), patients had significantly smaller volumes in all but one analyses (left banks of superior temporal sulcus) (Suppl. Table 3).

|  | **Age-, sex- and scanner-adjusted ANCOVAs**  **(Patients with SMI vs HC)** | | |
| --- | --- | --- | --- |
|  | **Direction** | **P-values** | **Partial eta-squared** |
| **TGMV** | -^1^ | <0.001 | 0.013 |
| **Cortical volume** | - | <0.001 | 0.013 |
| **Left regional cortical volumes** |  |  |  |
| Caudal middle frontal | - | 0.006 | 0.008 |
| Precentral | - | 0.003 | 0.010 |
| Lingual | - | <0.001 | 0.013 |
| Middle temporal | - | 0.009 | 0.008 |
| Banks of superior temporal sulcus | - | 0.267 | 0.001 |

**Suppl. Table 3.** P-values and effect sizes (partial eta-squared) of age-, sex- and scanner-adjusted analyses of covariance (ANCOVAs) investigating main effects of diagnostic status (SMI/HC) on grey matter volumes that differed significantly between HSV1+ and HSV1- patients with SMI

^1^Smaller grey matter volumes in patients with SMI compared with HC

***IQ analysis***

*Patients with severe mental illness*

The residuals of the overall model were approximately normally distributed (approximately symmetric to moderately left-skewed, skewness -0.487) determined by visual inspection. There were no outliers with studentized residuals greater than three standard deviations. There was a deviation from the homogeneity of variances assumption assessed with Levene’s test (p=0.032) indicating unequal variances. We computed bootstrapped bias-corrected and accelerated (BCa) 95% confidence intervals (CI). As in the model reported in the main text, there was still a statistically significant difference in IQ between HSV1+ and HSV1- (BCa 95% CI, 0.563 to 6.148), p=0.019.

*Healthy controls*

There was homogeneity of variances evaluated with Levene’s test, p=0.510. The residuals of the overall model were approximately normally distributed determined by visual inspection. There were three outliers with studentized residuals greater than three standard deviations. These cases did not exhibit high leverage (0.01 for all three cases) or high Cook's distance value (0.03, 0.03 and 0.02, respectively) and were included in the final model. In a model where these cases were excluded, there was still no significant main effect of HSV1 status on IQ (p=0.072).

***TGMV and IQ analysis in diagnostic subgroups***

|  | **HSV1+** | | **HSV1-** | |  |
| --- | --- | --- | --- | --- | --- |
|  | **N^1^** | **Mean (SD) or %** | **N^1^** | **Mean (SD) or %** | **P value^2^** |
| **SZ spectrum**  **(48.1% seropositivity)** |  |  |  |  |  |
| Sex (% women) | 115 | 41.7 | 124 | 42.7 | 0.875 |
| Age (years) | 115 | 31.6 (9.2) | 124 | 30.5 (9.5) | 0.386 |
| Education years | 115 | 12.5 (2.5) | 124 | 12.8 (2.6) | 0.429 |
| Handedness  (% right-handedness) | 115 | 88.7 | 124 | 88.7 | 0.977 |
| DOI (years) | 113 | 7.9 (6.7) | 119 | 7 (7.5) | 0.324 |
| PANSS total score | 113 | 59.3 (16) | 122 | 59.4 (18.5) | 0.949 |
| GAF-S | 115 | 45.4 (13.1) | 124 | 45 (12.6) | 0.822 |
| GAF-F | 115 | 46.1 (12.4) | 124 | 46.2 (13) | 0.940 |
| On antipsychotics (%) | 115 | 87.8 | 124 | 85.5 | 0.595 |
| On antidepressants (%) | 115 | 33.9 | 124 | 30.6 | 0.589 |
| On antiepileptics (%) | 115 | 17.4 | 124 | 12.1 | 0.247 |
| On lithium (%) | 115 | 2.7 | 124 | 1.6 | 0.674^3^ |
| AUDIT score | 81 | 7.3 (7.1) | 91 | 7.7 (6.7) | 0.683 |
| DUDIT score | 84 | 4.8 (8.4) | 92 | 5.1 (8.7) | 0.788 |
| ICV (cm^3^) | 115 | 1564 (199) | 124 | 1607 (168) | 0.074 |
| **BP spectrum**  **(45.3% seropositivity)** |  |  |  |  |  |
| Sex (% women) | 82 | 53.7 | 99 | 59.6 | 0.422 |
| Age (years) | 82 | 36 (11.7) | 99 | 32.9 (11.3) | 0.066 |
| Education years | 81 | 13.7 (2.2) | 99 | 13.4 (2.3) | 0.465 |
| Handedness  (% right-handedness) | 81 | 85.2 | 99 | 90 | 0.337 |
| DOI (years) | 82 | 14.7 (10.7) | 99 | 12 (9.5) | 0.075 |
| PANSS total score | 81 | 44.7 (8.9) | 98 | 45.6 (10.4) | 0.537 |
| GAF-S | 82 | 56.4 (10.5) | 99 | 57 (10.6) | 0.708 |
| GAF-F | 82 | 53.6 (12) | 99 | 54.7 (11.7) | 0.553 |
| On antipsychotics (%) | 82 | 54.9 | 99 | 51.5 | 0.652 |
| On antidepressants (%) | 82 | 31.7 | 99 | 41.4 | 0.178 |
| On antiepileptics (%) | 82 | 46.3 | 99 | 33.3 | 0.074 |
| On lithium (%) | 82 | 18.3 | 99 | 20.2 | 0.746 |
| AUDIT score | 58 | 9.1 (7.5) | 58 | 7.8 (5.3) | 0.748^4^ |
| DUDIT score | 60 | 3.7 (7.6) | 63 | 2.3 (5.6) | 0.367^4^ |
| ICV (cm^3^) | 82 | 1573 (155) | 99 | 1569 (167) | 0.438 |

**Suppl. Table 4.** Group differences between herpes simplex virus 1 (HSV1) immunoglobulin G (IgG) seropositive (HSV1+) and seronegative (HSV1-) patients with schizophrenia (SZ) and bipolar (BP) spectrum disorders in sex, age, education years, handedness (right-handedness vs. left-handedness/ambidexterity), duration of illness (DOI), Positive and Negative Syndrome Scale (PANSS) total score, the Global Assessment and Functioning-symptoms (GAF-S) and GAF-functioning (GAF-F) scores, the percentage of patients on antipsychotics, antidepressants, antiepileptics and lithium, alcohol use disorder identification test (AUDIT), drug use disorder identification test (DUDIT) score and estimated total intracranial volume (ICV). Patients with SZ and BP spectrum disorders did not significantly differ in HSV1 seropositivity, p=0.567, assessed with chi-square test

^1^Number of participants with data in each variable

^2^Chi-square test or t-test

^3^Fisher’s exact test

^4^Mann-Whitney U test

***Levene’s tests for the post-hoc analyses***

Post-hoc analyses/Analysis of cortical, subcortical and cerebellar grey matter volumes: HSV1 status on cortical grey matter volume: p=0.616; HSV1 status on subcortical grey matter volume: p=0.827; HSV1 status on cerebellar grey matter volume: p=0.362; HSV1 status on left caudal middle frontal**,** left precentral, left lingual, left middle temporal and left banks of superior temporal sulcus volumes: p=0.748, 0.636, 0.831, 0.562 and 0.246, respectively. Post-hoc analyses/Total grey matter volume and IQ analysis in diagnostic subgroups: HSV1 status on TGMV in SZ spectrum: p=0.936; HSV1 status on TGMV in BP spectrum: p=0.972; HSV1 status on IQ in SZ spectrum: p=0.429; HSV1 status on IQ in BP spectrum: p=0.131.

**References**

1 Andreou, D., Jorgensen, K. N., Wortinger, L. A., Engen, K., Vaskinn, A., Ueland, T. *et al.* Cytomegalovirus infection and IQ in patients with severe mental illness and healthy individuals. *Psychiatry Res* **300**, 113929, doi:10.1016/j.psychres.2021.113929 (2021).

2 Andreou, D., Jorgensen, K. N., Nerland, S., Engen, K., Yolken, R. H., Andreassen, O. A. *et al.* Cytomegalovirus infection associated with smaller dentate gyrus in men with severe mental illness. *Brain Behav Immun* **96**, 54-62, doi:10.1016/j.bbi.2021.05.009 (2021).

3 Faul, F., Erdfelder, E., Lang, A. G. & Buchner, A. G*Power 3: a flexible statistical power analysis program for the social, behavioral, and biomedical sciences. *Behav Res Methods* **39**, 175-191, doi:10.3758/bf03193146 (2007).
